# Enzyme-Based Electrochemical Biosensors for Microfluidic Platforms to Detect Pharmaceutical Residues in Wastewater

**DOI:** 10.3390/bios9010041

**Published:** 2019-03-15

**Authors:** Ana Lucia Campaña, Sergio Leonardo Florez, Mabel Juliana Noguera, Olga P. Fuentes, Paola Ruiz Puentes, Juan C. Cruz, Johann F. Osma

**Affiliations:** 1 Department of Electrical and Electronics Engineering, Universidad de los Andes, Cra. 1E No. 19a-40, Bogotá, DC 111711, Colombia; al.campana10@uniandes.edu.co (A.L.C.); sl.florez10@uniandes.edu.co (S.L.F.); mj.noguera10@uniandes.edu.co (M.J.N.); op.fuentes@uniandes.edu.co (O.P.F.); 2 Department of Biomedical Engineering, Universidad de los Andes, Cra. 1E No. 19a-40, Bogotá, DC 111711, Colombia; p.ruiz@uniandes.edu.co (P.R.P.); jc.cruz@uniandes.edu.co (J.C.C.)

**Keywords:** enzymes, biosensors, pharmaceutical residues, electrochemistry, microfluidics

## Abstract

Emerging water pollutants such as pharmaceutical contaminants are suspected to induce adverse effects to human health. These molecules became worrisome due to their increasingly high concentrations in surface waters. Despite this alarming situation, available data about actual concentrations in the environment is rather scarce, as it is not commonly monitored or regulated. This is aggravated even further by the absence of portable and reliable methods for their determination in the field. A promising way to tackle these issues is the use of enzyme-based and miniaturized biosensors for their electrochemical detection. Here, we present an overview of the latest developments in amperometric microfluidic biosensors that include, modeling and multiphysics simulation, design, manufacture, testing, and operation methods. Different types of biosensors are described, highlighting those based on oxidases/peroxidases and the integration with microfluidic platforms. Finally, issues regarding the stability of the biosensors and the enzyme molecules are discussed, as well as the most relevant approaches to address these obstacles.

## 1. Introduction

Many places around the world currently face water pollution due to the increasing volumes of waste discharged to superficial waters. This is in turn, the result of intensified industrial, agricultural and household activities. Consequently, the world has witnessed a growing scarcity of potable water and a decrease of aquatic biodiversity. A number of treatment processes have been devised to tackle these issues, which are mainly based on a proper determination of physicochemical parameters, the presence of microorganisms, and the possible uses of the recovered waste [1,2,3,4]. A complete characterization of polluted water resources is still elusive mainly due to the absence of tools for in situ, rapid, and sensitive monitoring of field samples. Pharmaceutical derivatives are some of the most difficult contaminants to detect and remove from water. One of the main pharmaceutical products found in wastewater are antibiotics, which are generally originated from both human and animal therapeutic and nutraceutical preparations [5].

In recent decades, various methods have been reported for the detection of pharmaceutical waste in wastewater including optical methods, immunoassay tests, molecular spectroscopy-based techniques and devices that rely on optical fiber technology [6]. In general, these methods incorporate antibodies, antigens, and enzymes as active components for the detection of specific antibiotics. This is mainly done by tracking changes in the intensity of the reflected light and/or the fluorescence of conjugated markers that become active upon triggering specific recognition mechanisms [6]. Electrochemical strategies for the detection of pharmaceutical residues have been reported to be sensitive, selective, robust, efficient and rapid alternatives in wastewater. In general, these strategies rely on the measurement of changes in ions or electrons generated by enzymes or antibodies immobilized on electrodes [7,8,9]. Over the past few years, there has been increasing interest in simultaneously detecting several water contaminants. This has been intended to reduce analysis times and production costs of biosensors.

This chapter is dedicated to describe both electrochemical biosensors based on enzymes such as laccase and oxidase/peroxidase, and amperometric microfluidic biosensors. Additionally, we provide a set of recommendations for the design and testing of biosensor prototypes.

## 2. Enzymatic Biosensors Applied to Microfluidic Systems

**Figure 1:**
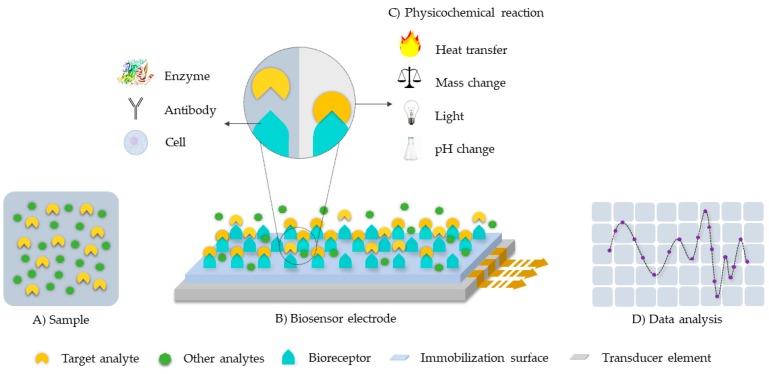
Schematic of a typical biosensor system architecture. The target analyte is detected by the bioreceptor and translated into a signal for analysis with the aid of a transducer. (**A**) Sample. (**B**) Biosensor electrode: composed by the bioreceptor, the immobilization surface and the transducer element. (**C**) Physicochemical reaction. (**D**) Data analysis.

Sensing platforms are generally composed of a recognition element and a transducing device as seen in Figure 1 [10,11,12]. In the particular case of biosensors, the recognition element is a biomolecule with stimuli-responsive capabilities. The transducing device converts, therefore, a measurable biological response or physicochemical change into an electrical signal [11,12]. Among the most common recognition molecules are: enzymes, antibodies, phages, or single-stranded DNA [10,11,13]. The recognition element in affinity biosensors binds permanently or semi-permanently with the analyte of interest, while for catalytic biosensors, the element maintains a non-permanent interaction in the form of a chemical reaction or electron transfer [10].

Enzyme-based sensors are highly specific catalytic biosensors where the recognition elements are extremely selective enzyme molecules immobilized on transducing surfaces known as electrodes [10,14,15]. Under the presence of a suitable substrate, the enzymes catalyze electrochemical reactions involving electroactive products or measurable electric changes on the transducer and the sample [15,16]. The concentration or redox potential of an analyte in a sample can be determined via measurements of potential, charge, or current. The devices to conduct these measurements apply potentiometric, impedimetric and amperometric techniques, respectively [14,16,17].

**Figure 2:**
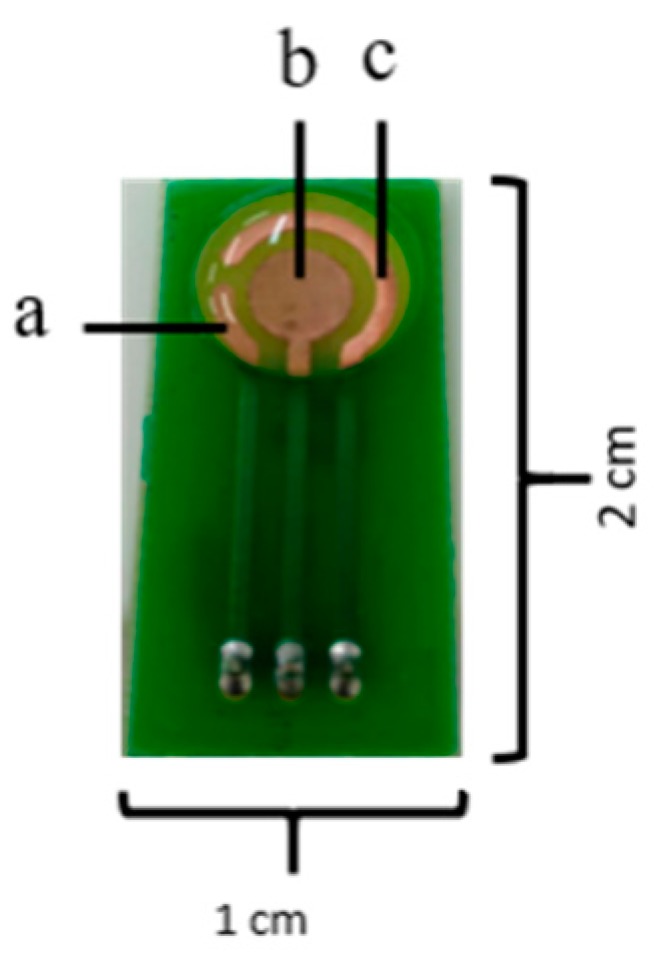
Three electrode sensor configuration. (**a**) reference, (**b**) working and (**c**) counter electrodes.

The amperometric methods are the most suitable to perform the transduction of the enzymatic response into a quantifiable signal [14]. In this case, the number of species involved in the redox process is related to the electrons generated when a fixed potential is applied between two electrodes, which can be ultimately detected as current change. Additionally, amperometric methods offer the possibility for real time detection, as well as ease for mass production [10,17,18]. The amperometric electrochemical biosensors can conduct measurements with the aid of two or three electrodes. In the two-electrode configuration, one functions as the working electrode while the other works as the reference. The sample is usually measured with the working electrode where the enzyme molecules are immobilized. Generated currents are evaluated by comparing the two electrodes. In the case of the three-electrode configuration, current flows between the working and a third electrode called counter while voltage is applied between the reference and the working electrodes (Figure 2) [19]. The surfaces of the electrodes are generally made of carbon and noble metals [17].

### 2.1. Immobilization Methods

**Figure 3:**
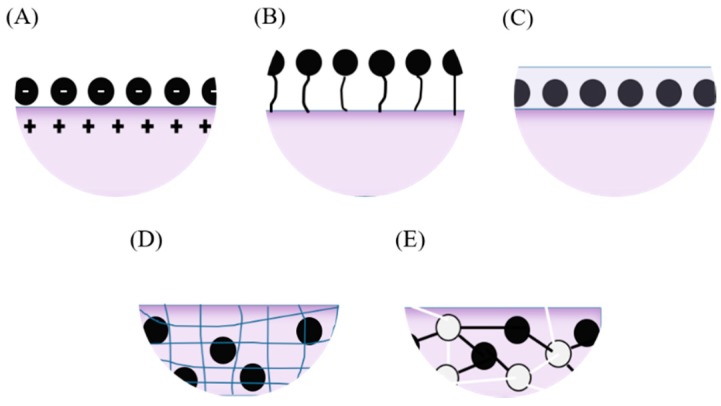
Enzyme immobilization onto electrodes. (**A**) Physically adsorbed through electrostatic interactions, (**B**) covalently bound to the surface, (**C**) entrapped within a film, (**D**) encapsulated within a porous surface and (**E**) cross-linked within the surface.

An essential procedure for the manufacture of electrodes in an electrochemical-enzymatic biosensor is the immobilization of enzyme molecules [16,20]. This can be achieved via physical adsorption, covalent binding, entrapment, encapsulation or cross-linking (Figure 3) [16,18]. Immobilization not only helps to prolong the shelf life of enzymes over time but also reduces the enzymatic time response. The process of immobilization of laccase molecules on electrode surfaces plays a key role in the performance of the biosensors. This is because during immobilization, the enzyme molecules are prone to conformational changes that might ultimately lead to partial or total loss of activity [18,21,22,23,24,25]. To enable these methods, activation of functional groups on the support surface might be required prior to immobilization to assure high immobilization yields [21,22].

When immobilizing laccase molecules via adsorption, surface attachment is achieved by the interplay of Vander Waals forces, hydrogen bridges, and hydrophobic, hydrophilic or ionic interactions [21,22,24]. In the case of covalent immobilization, the enzyme molecules react with the activated functional groups on the surface to form strong covalent bonds [22,25]. This immobilization approach generally leads to high conformational stability for the immobilized molecules [21]. Unfortunately, a risk of permanently damaging the active site of the enzyme molecules exists [25]. Moreover, under certain conditions, this approach has been reported to produce self-assembling, which might lead to detrimental intramolecular bonding. Some of the crosslinkers responsible for this include homo- and hetero-bifunctional molecules such as (3-Aminopropyl)triethoxysilane (APTES) and Glutaraldehyde [22,25]. In the case of entrapment, laccase molecules are trapped into a polymeric matrix to try to maintain their structural stability [22,25]. Encapsulation works by confining the enzyme molecules into semipermeable polymeric microspheres [22,25]. Finally, in the electrochemical immobilization, the enzyme molecules are oxidized by applying an electric potential difference to induce electron losses that are ultimately compensated by forming bonds with the support surface [18] as seen in Table 1.

The interaction between a target molecule in the sample and a specific enzyme is fairly well approximated by the Michaelis-Menten equation [14,16,20] (Equation (1)):

(1) $$V_{0}=\frac{V_{max}\;\left[S\right]}{K_{m}+\left[S\right]}$$

Rates of reaction ($V_{0}$) are experimentally obtained at different substrate concentrations ([S]) as described by the kinetics equation where $V_{max}$ is the reaction rate at enzymatic saturation concentration and $K_{m}$ is the enzyme-substrate affinity. A good enzymatic biosensor seeks to be sensitive enough to show high $V_{0}$ values in response to low substrate concentrations.

After immobilization, the amount of conjugated enzyme can be estimated by cyclic voltammetry analysis (CV), electrochemical impedance spectroscopy (EIS), Fourier transform infrared (FTIR) spectroscopy, and atomic force microscopy (AFM) [18,26]. For the case of CV and EIS, immobilization is demonstrated if a characteristic electron transfer from laccase catalytic turnover is observed [18]. FTIR spectroscopy confirms covalent binding of enzymes and crosslinkers by collecting information on the surface functional groups. Topography features via AFM allow to collect information on the surface disposition of the enzyme molecules and possible clustering processes during immobilization [26]. At the same time, it is important to evaluate the long-term stability of the enzyme-surface attachment to determine whether the biosensor can be reused and possibly be incorporated into a cyclic operation.

The correct operation of the biosensor is evaluated by characterizing the response signal in the presence of commercially available oxidizing enzyme substrates. A concentration curve is built for each substrate to determine the limit of detection, the signal stability upon concentration changes, the sensitivity range of the device, and the repeatability. In the case of Laccase, oxidizing standards are 2,2′-azino-bis(3-ethylbenzothiazoline-6-sulphonic acid) (ABTS) and syringaldazine [20,27,28,29]. Selectivity can be probed by calculating the selectivity coefficient, which can be recovered from concentration curve analysis of possible interference analytes at a constant concentration of substrate [30,31].

Operational stability of the biosensor can be estimated by conducting measurements with the device under extreme conditions of pH and temperature [20,30] Storage stability assures the robustness of the sensor response in the long-term. Optimal storage conditions strongly depend on the type of immobilization approach and the variability of the environmental conditions [22,31,32].

The enzymatic component of the biosensor is perhaps the most sensitive to large swings in operational conditions. To tackle this issue some of the strategies include chemical modifications of pendant groups on the enzyme surface, conjugation with stabilizing polymer molecules, and activation via increased hydrostatic pressure [21].

### 2.2. Device Prototyping and Testing

New strategies and methods for the development of electrochemical biosensors rely on microfabrication techniques and integrated electronics. The portability and ultra-low volume requirements of microfluidic analytical devices reduce sample manipulation, transfer, and storage; that ultimately, help prevent contamination [18,33]. Understanding momentum transport phenomena for fluids inside the microfluidic systems is the key to define proper manufacturing processes and protocols. In the section below, we discussed the equations that govern these phenomena.

#### 2.2.1. Microfluidics Fundamentals

The conservation of mass over time within a system is described by the continuity equation (Equation (2)):

(2) $$\frac{d\rho }{dt}+\frac{d\left(\rho u\right)}{dx}+\frac{d\left(\rho v\right)}{dy}+\frac{d\left(\rho w\right)}{dz}=0$$

where ρ is the density of the fluid and u, v and w are the components of the velocity in *x*, *y* and *z*, respectively. A conventional simplification for Equation (2) is to consider that the system is at a steady state, which means the system is invariant on time [34,35]. Additionally, if the fluid is considered incompressible (ρ = constant), then the expression can be simplified to Equation (3).

(3) $$\frac{du}{dx}+\frac{dv}{dy}+\frac{dw}{dz}=0$$

Conservation of momentum transfer led to the Navier-Stokes equations (Equation (4)), which can be solved along with (Equation (3)) to find the velocity profile of the fluid flowing within a microfluidic system.

(4) $$\rho \left[\frac{\partial V}{\partial t}+\left(V\cdot \nabla \right)V\right]=-\nabla P+\rho g+\mu \nabla^{2}V$$

where *V* is the velocity vector, *P* is the pressure, *g* is the gravitational field and *μ* is the viscosity of the fluid [34,35,36]. Due to the small scale of channels in microfluidic systems, gravitational forces are negligible. Furthermore, in microfluidics viscous forces dominate over convective forces. This can be seen with the aid of the Reynolds number (*Re*), which is defined according to the relationship in Equation (5).

(5) $$Re=\frac{\mathrm{convective}\;\mathrm{forces}}{\mathrm{viscous}\;\mathrm{forces}}=\frac{\rho V_{m}D_{h}}{\mu }$$

where $D_{h}$ is the hydraulic diameter, is the density of the fluid, is the dynamic viscosity, and $V_{m}$ is the mean velocity. In the case of microfluidics, the Re numbers are typically below 1 (*Re* < 1) [37]. This flow regime is known as a laminar and under this condition; the convective terms of Equation (4) are negligible. Finally, if the steady state is assumed, Equation (5) can be simplified to:

(6) $$-\nabla P+\mu \nabla^{2}V=0$$

**Figure 4:**
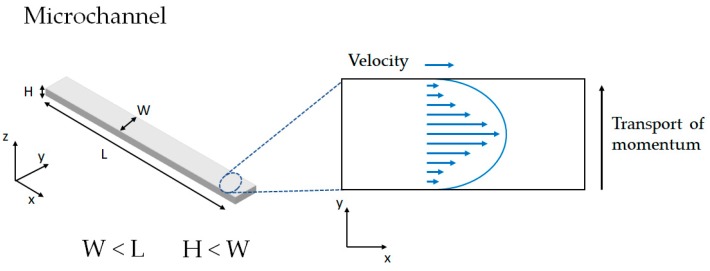
Example of momentum transport and velocity profile generated in a microfluidic channel.

If for instance, we considered a microfluidic system with squared channels, it is conventional to find that the width of the channels (W) is considerably smaller than the length of the channels (L). Likewise, the height of the channel (H) is shorter than W (Figure 4). Under these geometry considerations, the problem can be considered unidimensional with molecular momentum transfer only in the y direction (Equation (7)):

(7) $$-\frac{dP}{dx}+\mu \frac{d^{2}u}{dy^{2}}=0$$

By solving Equation (7) it is possible to determine the required inlet and outlet pressures on the channel to generate a specific velocity profile in the fluid [34,37].

#### 2.2.2. Prototypes Circuit Approximation

**Figure 5:**
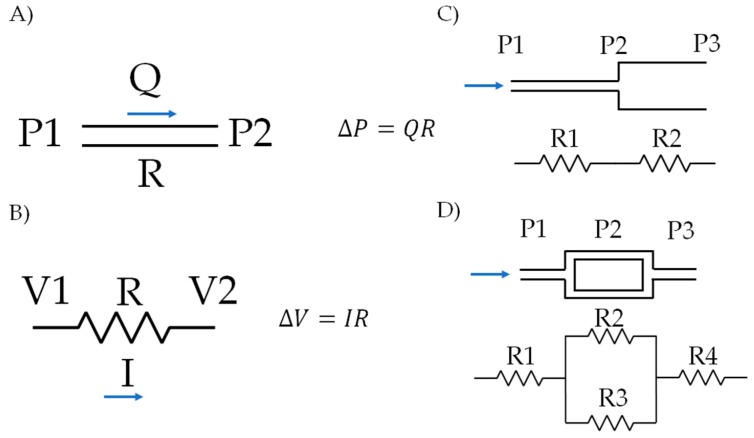
Circuit approach for modeling fluid flow in microsystems: (**A**) pressure and flow ratio in a microchannel, (**B**) relationship for voltages and currents in a circuit, (**C**,**D**) examples of different microsystems configurations and their corresponding circuit analog.

An easy and quick way to estimate the correlation between the pressure gradient, flow rate and resistance for a fluid within a microfluidic system is by using an electrical equivalent approach. To solve the equations, one can consider an analogy between the parameters in fluid mechanics and those of the electrical systems. Accordingly, the flow rate (Q) within the microsystem is due to a change in pressure (P) between inlets and outlets, which correspond to current flow (I) and the voltage potential (V) in circuits. In the two cases, an extra parameter must be considered, the resistance (R), which is understood as a restraining imposed by a system or material to the flow of fluids or electrons [34,35]. Figure 5 shows an example of different configurations of microsystems and the corresponding circuit analogy.

If we consider a microchannel with a circular cross-sectional area, the fluid flow equations are solved with the aid of the Hagen-Poiseuille relationship (Equation (8)):

(8) $$Q=\frac{AR^{2}}{8\mu L}\Delta P$$

where *A* is the cross-sectional area perpendicular to fluid flow, *R* is the radius of the channel and *L* is the length of the channel. Manufacturing of microfluidics usually leads to channels with geometries far from circular and for that reason, Equation (8) no longer applies. A modified version of Equation (8) includes the hydraulic radio ($r_{h}$) (Equation (10)), which is defined according to the expression in Equation (9):

(9) $$Q=\frac{{{Ar}_{h}}^{2}}{8\mu L}\Delta P$$

(10) $$r_{h}=\frac{2A}{\bar{p}}$$

where $\bar{p}$ is the perimeter of the cross-sectional area. This approach allows to consider the fluid flow in microfluidic channels with any cross-sectional area. With this new parameter, we can now rewrite Equation (8) and subsequently define the hydraulic resistor (Equations (11) and (12)):

(11) $$R_{h}=\frac{8\mu L}{{{Ar}_{h}}^{2}}$$

(12) $$\Delta P=QR_{h}$$

The mass balance for the system is also achieved if we consider that for the electric circuit analogy, the sum of the currents in each node must equal zero. This leads to Equation (13) for the system:

(13) ∑Q=0

This simple yet powerful approach is always used by us to estimate changes of pressure and flow within a microsystem without necessarily knowing the details of velocity distribution inside it [35].

#### 2.2.3. Mixing and Separation

Some of the most common applications of microfluidic systems include separation and mixing processes, which are useful to concentrate or remove some components of the analyzed mixtures. Depending on the application, it is possible to separate micrometer (e.g., cells) to nanometer size (e.g., proteins) particles from complex mixtures. Separation of particles with significant differences in sizes or densities, generally rely on devices capable of generating sufficient inertial forces [38,39]. This can be achieved by spiral geometries because in this case the drag and lift forces acting on the particles force them to separate from each other within the microchannel. The separation efficiency strongly depends on properties of the particles such as mass, size, and density. Another efficient way to separate particles or molecules is by means of aqueous two-phase systems (ATPS) [39,40]. These systems take advantage of the electrostatic potential at the interface between two immiscible liquids and the tendency of charged molecules to preferentially partition into one of the fluids in contact [40,41]. The net superficial charge can be also exploited for separation by making use of electrophoretic mechanisms. This is also the case when the molecules of interest exhibit magnetism, as they can be separated via magnetophoresis [42,43].

A major challenge when working with microfluidic systems is the difficulty to generate efficient mixing patterns mainly due to the low Reynolds numbers (*Re* < 1). To tackle this issue, it is necessary to generate turbulence through special geometries such as Zigzag channels, 3-D serpentine structures, and twisted channels [44]. Micromixing can be achieved either through active mixing or passive mixing [45,46]. In the case of active mixing, integration of components is accomplished via an external energy source such as ultrasound, acoustic vibrations, small impellers, or electrokinetic instability. In contrast, passive micromixing requires a device to intimately put in contact the components through disturbances in the mixing patterns. These devices include chaotic flow configuration, flow recirculation configuration, colliding jet, split and recombine flow configurations.

#### 2.2.4. Simulations

An easy and economical way to make and test prototypes is by Computational Fluid Dynamics (CFD) simulations. There are different software packages to implement CFD simulations, one of which is Comsol Multiphysics^®^. This software solves partial differential equations (PDE) associated with physical phenomena via the finite element method (FEM) [47]. In this approach, PDE equations are solved by putting the functions of interests in the integral form, where the unknowns are discretized as summations over the functions defined on the finite elements. Depending on the topology of the geometry, i.e., 2D or 3D, the elements are assembled into polygonal forms, thereby creating a mesh over the computational domain. The FEM is adaptable to complex geometries and can handle discontinuous gradients of a variable. Nevertheless, like any other discrete numerical method for solving continuous PDE, it introduces some numerical error. This can be minimized by varying the meshing approach or by refining the mesh on those spots where the flux of gradients is the greatest [47].

**Figure 6:**
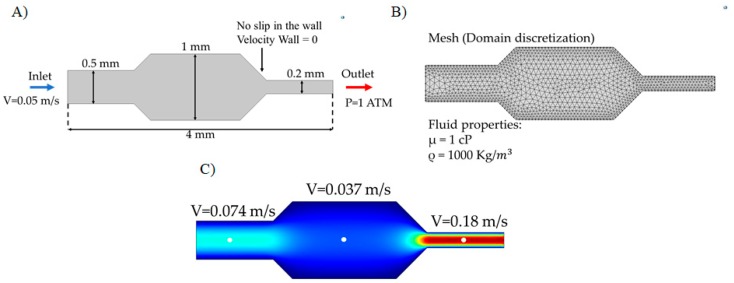
Simulation of microfluidic system in Comsol Multiphysics ^®^ (**A**) Simulation domain with the corresponding boundary conditions (inlet speed, outlet pressure and non-slip in the walls), (**B**) discretization of the domain by a meshing process and the list of physical properties of the fluid, (**C**) velocity profile in the channel as recovered from Computational Fluid Dynamics (CFD) simulations.

Figure 6 shows an example of the simulated fluid flow in a microfluidic channel after implementation in the CFD module of Comsol Multiphysics^®^. The Navier-Stokes equations were solved to find the velocity distribution (see above). Maximum velocity values are shown for each section of the microchannel in Figure 6C. Also, it is possible to recover data on pressure changes within the system, shear stress, and shear rate.

This simulation tool has been used in different studies to optimize the response of different types of biosensors [48,49,50]. This optimization can be from the operation values of the biosensor (voltages, resistance values, flow rates, etc.) or changes in the geometry.

#### 2.2.5. Fabrication of Prototypes

**Figure 7:**
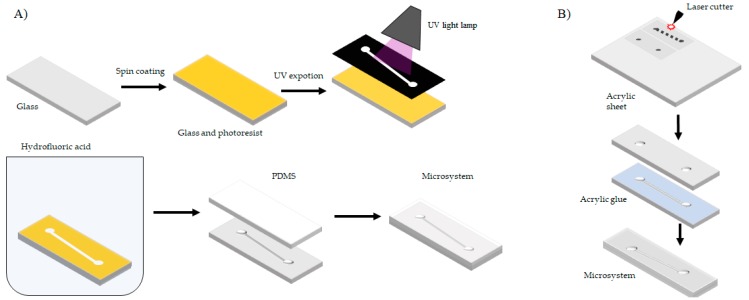
(**A**) Manufacturing process by photolithographic techniques and (**B**) manufacturing process by laser cutting.

Chemical etching is based on eroding a surface to generate microchannels with depths ranging from 5–15 µm [51,52]. Glass is the preferred substrate to manufacture microsystems by this technique [53]. Chemical etching on glass starts by covering the surface with a sacrificial layer known as photoresist by means of a spin coating machine. Depending on the properties of the photoresist and the speed of the spin coating process, different sacrificial layer thicknesses can be generated. Next, the photoresist covered glass sheet is exposed to a mask with a layout of the microsystem. Subsequently, the two surfaces (photoresist and glass) are immersed in hydrofluoric acid. In areas with absence of photoresist, the glass surface is eroded, thereby generating the microchannel. Finally, the microchannel is sealed with a layer of PDMS [54]. Figure 7A shows a schematic of the etching process. While this technique offers a high resolution, the involved costs are relatively high due to the multistage processing required.

Physical etching is based on making laser cuts and laser eroding of surfaces to achieve channels with depths in the range of 1–0.3 mm. The preferred material to apply this technique is Poly(methylmethacrylate) (PMMA) [55,56]. To obtain a PMMA sheet with the desired depths, surface erosion and cuts are conducted with the aid of a programmed laser beam. Then the cut pieces are glued together with an acrylic adhesive and maintained under constant pressure for several hours. Figure 7B shows a schematic of the laser cutting process. This technique is inexpensive, simpler and faster when compared with wet etching, soft lithography and thermoforming [57]. Laser cutting precision and fidelity is limited when working below 0.5 mm.

## 3. Oxidase/Peroxidase Based-Biosensors

**Figure 8:**
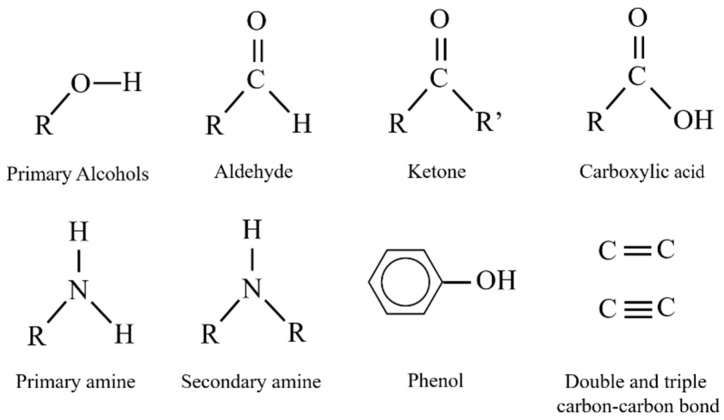
Functional groups catalyzed in redox reactions by oxidase and peroxidase enzymes.

A family of enzymes with a well-recognized ability for the detection of substances including pharmaceuticals are oxidases and peroxidases [58,59]. They have been reported to work either independently or in combination. Through oxidation-reduction reactions (REDOX), these enzymes catalyze the biotransformation of numerous compounds (Figure 8) [60,61,62].

Oxidase/Peroxidase based-biosensors have been successfully used to detect molecules such as glucose, alcohol, putrescine, oxygen, hydrogen peroxide and other small metabolites. This has been useful for applications in the food industry, medical diagnosis and in vitro assays of pharmaceutical trials [21,59,63,64,65]. Even though these enzymatic biosensors exhibit high sensitivity and selectivity toward the detection of small molecules, their use is limited due to issues regarding low stability of the enzyme molecules during the fabrication, special storage conditions, and complicated implementation protocols [63]. One avenue to overcome these obstacles is to immobilize the enzyme molecules onto the device surfaces. This approach has been reported to enhance operational and storage stability, sensitivity, selectivity, response time and reproducibility [66].

Physical adsorption was the immobilization approach used by Somasekhar et al. to develop an amperometric biosensor for alcohol detection [30]. In their contribution, a protein matrix of alcohol oxidase (AOx) with entrapped ferrocene was prepared via a combination of microwaves and physical adsorption on a sol-gel chitosan system [30]. Covalent binding has also been used in the fabrication of glucose biosensors to co-immobilize glucose oxidase (GOx) and HRP into polyacrylic acid deposited on a transmission electron microscope (TEM) grid with the aid of EDC and NHS as crosslinkers [67]. The same approach was also implemented in a biosensor for putrescine detection. In this case, putrescine oxidase/peroxidase was co-immobilized via glutaraldehyde crosslinking on a Ketjen Black mesoporous electrode [68].

In addition, the electrical responses of Oxidase/Peroxidase amperometric biosensors to a specific substrate can be measured either by direct or indirect methods. Hervás et al. implemented an indirect method to measure glucose by correlating it with the amount of H_2_O_2_ released by the GOx molecules immobilized into polymethacrylic acid microparticles. The released H_2_O_2_ was detected upon oxidation on an electrode by measuring the produced current changes [69]. In contrast, a direct method is illustrated by direct electron transfer in an amperometric alcohol biosensor coupled with a putrescine detector. The system consists of an electroactive surface for facile electron exchange made of multi-walled carbon nanotubes. In this case, electrons transfer between the modified glassy carbon electrode and the HRP for H_2_O_2_ reduction [30]. Putrescine oxidation by putrescine oxidase was coupled to the reduction of H_2_O_2_ by putrescine peroxidase while electrons needed for the reaction are provided by the electrode [68]. The PANI film within the Halal verification biosensor provides not only electrical responses but colorimetric changes to the presence of ethanol in beverages. This is accomplished by a redox reaction with H_2_O_2_ that promotes a color change from green to blue [31]. Detection of glucose has also been achieved qualitatively by changes in the optical appearance of 4-cyano-40-pentylbiphenyl. This is attributed to a molecular orientation change from planar to homeotropic when increasing amounts of glucose are present [67].

The most commonly found pharmaceutical residues are antibiotics, analgesics, anti-inflammatories, steroid hormones, antihypertensives, and antidepressants, which originate from both human and veterinary therapeutics [70,71]. Oxidase/Peroxidase biosensors have become increasingly popular for detecting pharmaceutical compounds due to the sensitivity of these enzymes to such molecules [72]. Analgesics such as paracetamol have been detected by the HPR-mediated oxidation of the compound, as reported by Narang et al. [73] HPR immobilized on core-Shell ZrO@ Fe_3_O_4_ nanoparticles and embedded on a chitosan hybrid film were electrodeposited on Au electrodes for detection of paracetamol via cyclic voltammetry. Other enzyme-based biosensors have been reported for the detection of antibiotics such as penicillins and tetracyclines by means of peroxidases, laccases, and tyrosinases as the recognition elements [5,73].

## 4. Laccase Based-Biosensors

Laccase (EC 1.10.3.2, p-benzenediol: oxygen oxidoreductase) is an oxidoreductase enzyme, capable of oxidizing phenolic compounds into phenoxyl radicals, with the aid of the 4 copper electrons in its structure and the presence of molecular oxygen [27,74]. These enzymes are produced principally by fungi but they have been reported to be present in plants and some bacteria as well [75,76]. Due to their potent catalysis capabilities, laccases are widely used in the synthesis of organic compounds [28] and bioremediation of wastewater effluents. They have been indeed tested in the remediation of effluents from pharmaceutical manufacturing, production of textiles, paints, and logging [22,23].

This case analyzes biosensors for the detection and/or quantification of phenol compounds where the bioreceptor is the laccase enzyme. The chemical or physical reaction, called output response, needs to be converted to a measurable electronic signal by a transducer element (Figure 1) [77,78]. To achieve a selective detection, the bioreceptor needs to have a great affinity for the desired analyte, namely the phenol compounds [22].

According to the transduction method, biosensors can be categorized into electrochemical, optical and thermal [22,24].

**Figure 9:**
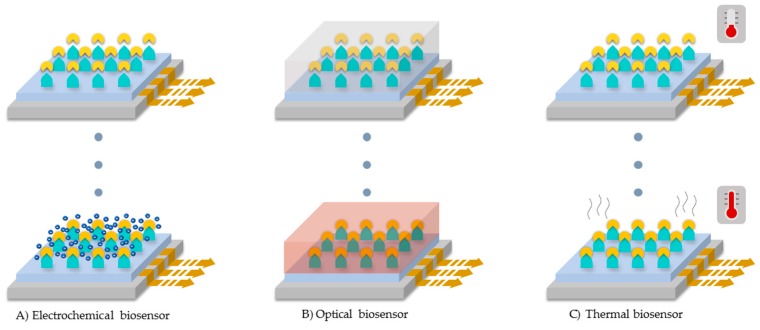
Laccase-based biosensors categories according to the transduction method used. (**A**) Electrochemical biosensor, (**B**) optical biosensor, and (**C**) thermal biosensor.

In general, electrochemical biosensors measure the electric potential difference or current due to electrons produced by the oxidation of analytes upon catalysis. As a result, molecular oxygen present in the medium is reduced to produce water molecules (See Figure 9A) [20,22,24]. Depending on the detection system used in the electrode, this type of biosensors can also be classified by the electrical magnitude at which the potential difference is measured [22,24]. The first type is termed voltammetric sensors in which the current flow passing through an electrode is measured as a response to an applied electric potential. In this case, the analyte-enzyme reaction is performed at a specific potential range where current changes are observable [22,24]. A special case of voltammetric sensors are the amperometric sensors where the potential is kept at a constant value [18,22,24]. The second type is the conductometric sensors, which analyze changes in the conductance of the sample medium during redox reactions. The third type is the potentiometric sensors, which incorporate two non-polarizable electrodes to measure the potential difference, the two electrodes are called working and reference [22,24].

Optical biosensors measure changes in spectroscopic properties of enzymatic catalysis products such as fluorescence and/or absorption. Some of the spectroscopic techniques include UV-VIS, FTIR, Raman and surface plasmon resonance, as seen in Figure 9B [22,25]. Finally, thermal biosensors measure enthalpy changes as a result of reactions conducted on the electrode surface (See Figure 9C) [22].

Table 1 shows the latest developments in biosensors for measuring analytes common to the pharmaceutical industry. Some of these analytes are feedstocks for the manufacture of antibiotics, analgesics, steroids and antioxidants [22,24,75]. For example, adrenaline, dopamine, norepinephrine and L-DOPA are often incorporated as active compounds into process for the manufacture of neurotransmitters [79].

The vast majority of experimental trials for enzyme-based electrochemical biosensors have been conducted in artificial wastewaters where the contents of the substances to be measured are fully known. Real wastewater matrices contain, however, a large variety of compounds with unknown concentrations including alkylphenols, cresols, aniline 2,4-dichlorophenol, some catechols, and phenolic resins [103]. The origin of many of these compounds is attributed to industrial, agricultural and domestic waste [104]. The presence of these compounds is largely responsible for the reduced output electrical signals of this type of biosensors when dealing with real samples. Additionally, Zilly et al. reported disruption of laccases’ active sites due to the presence of NaCl and Na_2_SO_4_, which are thought to promote conformational changes and even the blockage of the electron transfer machinery [105,106]. Enzyme degradation has also been reported by the presence of nitrite, thiosulfate, and cyanide [25]. Finally, it is crucial to take into account that in situ monitoring of field samples might require analyses at extreme temperatures and pH values, which are generally responsible for detrimental conformational changes.

## 5. Concluding Remarks and Future Perspectives

In a broader perspective, when compared to the conventional methods, laccase-based biosensors have a tremendous potential for the detection of phenolic compounds in wastewater thanks to a fast and in situ sample analysis, cheaper cost per analysis and the possibility for “real time” measurements [22]. Despite these advantages, the implementation of laccase-based biosensors has been limited due to issues regarding enzyme stability, which are generally exacerbated by sudden changes in pH, temperature, and ionic force [21,25]. As a result, an area of increasingly growing interest is the development of novel immobilization strategies that rely on a deeper understanding of surface-protein and protein-protein interactions.

An additional challenge in the application of laccase-based biosensors is their limited ability to exclusively detect one of the compounds present in a mixture, and especially if they are all substrates of laccase. An avenue to overcome this issue is to incorporate surface modifications capable of preferentially immobilizing the enzyme molecules in a conformation/orientation that favors the interaction with only the molecules of interest. This is important for industrial applications where, by identifying each analyte separately, a focused intervention can be performed in an in-line and real-time manner. This approach has proven useful to improve the regio- and enantio-selectivity of various enzymes, as a direct consequence of increasing the rigidity of the active site. Active sites with suppressed flexibility show a significant reduction in their ability toward catalyzing different types of compounds thereby providing higher selectivities [107,108]. While increasing selectivity may be attractive for some applications, one might be cautious when altering the active site 3D conformation as the enzyme turnover may decrease considerably. This could potentially lead to a decline in the detection limits and sensitivity of the biosensing platforms.

The limited access to potable water and considering that industrialization has accelerated in emerging economies around the world increasing the amount of discharged residues, represents a difficult challenge for modern science and engineering. There is a growing concern with a class of wastewater residues known as Pharmaceuticals and Personal Care Products (PPCPs) as a major threat to human health. In this chapter, we reviewed the latest developments in electrochemical methods for the detection of pharmaceutical residues, and particularly those associated with enzyme-based biosensors. Also, we detailed amperometric systems coupled to microfluidic platforms for sample processing. Most of the progress in the field of enzymatic biosensors is mainly due to the advancement of technologies to enable miniaturization of prototypes, ease of handling and portability, and the reduction of cost for field implementation. Despite the encouraging results, important drawbacks are yet to be addressed including low sensitivity and selectivity in multicomponent mixtures, low resilience to changes in pH and temperature, limited immobilization yields for the bioactive components, and largely constrained long-term and operational stability. These are commonly found samples in real field wastewater samples where complex mixtures usually contain more than one analyte of interest and at very low concentrations. These drawbacks can be overcome by implementing low-cost microfluidic devices capable of selectively separating and increasing the concentration of the analytes of interest prior to sensing.

Future developments include multienzyme portable devices capable of detecting several compounds at the parts per billion level in real-time. This approach is, however, challenging due to issues regarding proper surface orientation and high conformational stability for the immobilized enzyme molecules, limited long-term and operational stability, processing and deconvoluting complex data signals, providing reproducible and reproducible manufacturing protocols, and most critically—shorter response times. Research in the biosensors area should be compelled to manufacture faster, smaller and more efficient devices through the integration of electronics and biological systems. To meet this goal, biosensors may include the use of new integrated circuits that optimize miniaturization and profitability, with the implementation of wireless and Internet of Things (IoT) technology for remote control and data handling.

## Figures and Tables

**Table 1:** Laccase-based biosensors for pharmaceutical analytes as classified by the employed transduction and immobilization methods.

Transduction Method	Immobilization Method	Target Analyte	Measurement	Detection (Range| Limit| Time)	Characteristics (Selectivity | Stability)	Ref.
Electrochemical Biosensor	Adsorption	Adrenaline	Pt-BMI.PF6-Laccase Ag/AgCl reference electrode Pt wire as counter electrode	$9.99\times 10^{-7}∼2.13\times 10^{-4}$ M $2.93\times 10^{-7}$ M NS	Suitable (Untested) 20% loss of response after 90 days	[79]
		Epinephrine	Glassy carbon-GQDs-Laccase electrode Ag/AgCl reference electrode Pt wire as counter electrode	1∼120 µM 83 ηM NS	High (Against ascorbic acid, uric acid, cysteine, glutathione, tryptophan and a mix of all) NS	[80]
		L-Cysteine	Carbon-paste electrode Ag/AgCl reference electrode Pt wire as counter electrode	$1.97\times 10^{-4}∼3.24\times 10^{-3}$ M NS NS	High (Against hydroquinone and other inhibitors) Lifetime of 9 months (∼950 measurements) Maximum response at pH 7.0	[81]
		Dopamine	Laccase/(h-SiO_2_—PA)/Glassy-carbon electrode Saturated calomel reference electrode	0.99∼138.40 µM 0.17(±0.002) µM ∼1500 s	Good anti-interference ability 11% loss of response after 20 days	[82]
		Dopamine Adrenaline L-dopa Isoprenaline	Nujol/Graphite powder Laccase/Peroxidase as working electrode Ag/AgCl reference electrode Pt wire as counter electrode	D: $6.6\times 10^{-6}∼3.9\times 10^{-4}$ M A: $6.1\times 10^{-6}∼1.0\times 10^{-4}$ M L: $6.7\times 10^{-6}∼7.0\times 10^{-5}$ M I: $6.2\times 10^{-6}∼8.1\times 10^{-5}$ M D: $2.7\times 10^{-8}$ M A: $2.5\times 10^{-8}$ M L: $2.4\times 10^{-8}$ M I: $2.6\times 10^{-8}$ M 60 s	NS Lifetime of 2 months (∼500 measurements) D: Maximum response at pH 6.0 and 35 °C A: Maximum response at pH 7.0 and 35 °C L: Maximum response at pH 6.5 and 35 °C I: Maximum response at pH 6.0 and 35 °C	[83]
	Covalent binding	Pyrocatechol	Glassy-carbon electrode Ag/AgCl reference electrode Pt wire as counter electrode	3.98∼16.71 ηM 2.82 ηM NS	≤5% change in the response by environmental interferents 19% loss of response after 21 days	[84]
		Catechin	PDATT/Den(AuNPs) on glassy-carbon electrode Ag/AgCl electrode	0.1∼10 µM 0.05(±0.003) µM <10 s	NS 8% loss of response after 60 days Maximum response at pH 6.5 and 30 °C	[85]
		Norepinephrine	PDA-Laccase/Au-glucose dehydrogenase	0.5 ηM∼0.5 µM 0.07 ηM NS	NS 8.57% loss of response after 30 days	[86]
		Tartrazine	Laccase -AuNPs coated on a carbon-paste screen-printed electrode	0.2∼14 µM 0.04 µM 2 min	±10% change in the response by common coexisting substances 48.9% loss of response after 90 days Maximum response at pH 5.0	[32]
		Catechol	Graphite electrode Ag/AgCl reference electrode Pt wire as counter electrode	A: up to—2 mM B-C: up to—0.1 mM NS	NS A: 10 days, B: 30 days and C: 60 days of stability Maximum response at pH 5.0	[87]
		Catechol	Glassy carbon as working electrode Ag/AgCl reference electrode Pt wire as counter electrode	$3.2\times 10^{-6}∼1.96\times 10^{-5}$ M $2.07\times 10^{-6}$ M NS	NS	[26]
	Cross-linking	Acetaminophen Diclofenac	TiO_2_-Lac nanoparticles	NS	NS High stability at low pH values of 2–3 and 50–60 °C	[20]
		Pyrocatechol 1-naphthol o-phenylenediamine	Graphite electrode SCE as reference electrode Pt wire as counter electrode	0.6∼4.0 mM NS NS	NS Lifetime of 9 days (decreased response at longer times)	[88]
		2-amino phenol Catechol Pyrogallol Guaiacol	Clarke-type electrode (Au cathode and Ag/AgCl reference electrode)	0.5∼0.125 mM NS 60 s	NS 40% loss of response after 30 measurements Maximum response at pH 5.5–6.0	[89]
		Paracetamol: With/Without HBT	Dissolve oxygen electrode	HBT: 2∼15 µM W-HBT: 0.5∼3 µM 10 min	NS 0% loss of response after 7 h Maximum response at pH 4.5 and 35 °C	[90]
	Entrapment	Dopamine	Lac/Si/MWCNTs/ SPE electrode Ag/AgCl reference electrode	1.3∼85.5 µM 0.42 µM NS	High (Against ascorbic acid [AA]) 14% loss of response after 30 days	[91]
		Catechol	Nafion/Laccase-glassy carbon as working electrode Ag/AgCl reference electrode Pt wire for counter electrode	0.166∼7 µM 0.166 µM NS	≤3% change in the response by phenolic interferents 12.9% loss of response after 30 days	[92]
		Morphine	Clark oxygen electrode	3.2∼1000 µM A: 32 ηM N-A: 10 µM 1 min	High (Against codeine) NS	[93]
		Epinephrine	Laccase-carbon paste working electrode Ag/AgCl reference electrode Pt wire as counter electrode	4.98∼295 µM 1.84 µM NS	High (Against dopamine and phenol) 7.0% loss of response after 7 days	[94]
		Catechol	CNTs–CS/GC electrode Ag/AgCl reference electrode Pt wire as counter electrode	1.2∼30 µM 0.66 µM NS	NS <1% loss of response after 15 days	[95]
		Epinephrine Norepinephrine Dopamine	Os(PVI) 10-Laccase electrode Glassy carbon working electrode Ag/AgCl reference electrode Pt wire as counter electrode	NS E: 11 ηM N: 8 ηM D: 4 ηM 5 s	No selectivity between the catecholamines Lifetime of at least 1 month	[96]
Optical Biosensor	Adsorption	Catechol	Lac-polyacrylamide sensor film	L: $9.79\times 10^{-6}∼7.50\times 10^{-4}$ M H: $7.50\times 10^{-4}∼5.00\times 10^{-3}$ M NS 450 s	NS Lifetime of 30 measurements Maximum response at pH 5.0–6.0	[97]
		Adrenaline	Laccase-CuTAPc-Fe_3_O_4_-NPs	$2.0\times 10^{-7}∼9.0\times 10^{-7}$ M $1.0\times 10^{-8}∼9.0\times 10^{-8}$ M NS 30 s	NS 16% loss of response after 10 measurements	[98]
		Dopamine Norepinephrine Epinephrine	LacOF biosensor	NS D: 2.1 pg / mL N: 2.6 pg / mL E: 3.4 pg / mL 3 min	High (Against urine and plasma) <5% loss of response after 60 days	[99]
	Covalent binding	Adrenaline	Laccase-CuTAPc-Fe_3_O_4_ NPs	$2.0\times 10^{-7}∼9.0\times 10^{-7}$ M $1.0\times 10^{-8}∼9.0\times 10^{-8}$ M NS 30 s	NS 16% loss of response after 10 measurements	[98]
	Cross-linking	Catechol	Laccase-Hybrid Nafion/sol-gel silicate-MBTH film	0.5∼8.0 mM 0.33 mM 10 min	Suitable selectivity against Nafion/sol-gel silicate Lifetime of at least 2 months	[100]
		Catechol	Laccase-Au-Fe_3_O_4_ NPs	5.0∼70.0 µM 2 µM 40 min	NS Maximum response at pH 5.0	[101]
	Entrapment	Epinephrine Dopamine Norepinephrine	Liquid chromatography (HPLC) and detection by optical fiber (OF) coated with Laccase	5∼125 pg / mL E: 3.5 pg / mL D: 2.9 pg / mL N: 3.3 pg / mL 7 min	NS Lifetime of at least 2 months	[102]
Thermal Biosensor	Cross-linking	Phenol	Lac/PVA Microspheres	2∼8 mM NS NS	NS 13.7% loss of response after 100 days	[29]

* NS—not Specified.
